# STT3B promotes porcine epidemic diarrhea virus replication by regulating N-glycosylation of PEDV S protein

**DOI:** 10.1128/jvi.00018-25

**Published:** 2025-02-13

**Authors:** Huixin Zhu, Jinxiu Lou, Zhen Yang, Juan Bai, Ping Jiang, Xianwei Wang, Xing Liu

**Affiliations:** 1 Key Laboratory of Animal Disease Diagnostics and Immunology, Ministry of Agriculture, MOE International Joint Collaborative Research Laboratory for Animal Health & Food Safety, College of Veterinary Medicine, Nanjing Agricultural University 261674 , Nanjing, China; 2 Jiangsu Co-Innovation Center for Prevention and Control of Important Animal Infectious Diseases and Zoonoses, Yangzhou, China; University of North Carolina at Chapel Hill, Chapel Hill, North Carolina, USA

**Keywords:** PEDV, N-glycosylation, antiviral agents, STT3B, spike protein, viral replication

## Abstract

Porcine epidemic diarrhea virus (PEDV), a highly pathogenic enteric coronavirus, has caused significant economic losses worldwide in recent years. The PEDV spike (S) protein has been reported to undergo extensive N-glycosylation, suggesting that glycosylation plays a crucial role in PEDV replication. In this study, we demonstrated that the N-glycosylation pathway promotes PEDV replication by facilitating the glycosylation of the S protein. First, we observed that pharmacological inhibition of host N-glycosylation using specific inhibitors significantly reduces viral replication. Furthermore, genetic ablation of STT3A or STT3B, the catalytically active subunits of the oligosaccharyltransferase (OST) complex, revealed that the STT3B-OST complex, but not STT3A, is preferentially required for PEDV replication. Notably, we showed that the N-glycosylation of the PEDV S protein depends on the oligosaccharyltransferase activity of STT3B. Together, the study demonstrated the critical role of the N-glycosylation pathway in PEDV replication by elucidating the relationship between the N-glycosylation of the PEDV S protein and STT3B, thereby presenting a potential new target for the prevention and control of PEDV.

**IMPORTANCE:**

The highly N-glycosylated spike protein of porcine epidemic diarrhea virus (PEDV) is a multifunctional protein that plays a crucial role in the viral replication cycle. In this study, using pharmacological inhibitors, we demonstrated the importance of the N-glycosylation pathway in PEDV replication. Genetic analysis revealed that STT3B, one of the catalytically active subunits of the oligosaccharyltransferase complex, promotes viral proliferation by regulating the N-glycosylation of the PEDV spike protein. Our findings enhance the understanding of the role of the N-glycosylation pathway in viral infection and identify STT3B as a potential therapeutic target for controlling PEDV infection.

## INTRODUCTION

Porcine epidemic diarrhea virus (PEDV) is a highly pathogenic enteric coronavirus that causes severe diarrhea, vomiting, and dehydration. Swine of all ages can be infected with PEDV, and mortality in suckling piglets can be as high as 100% (1). PEDV is an enveloped, single-stranded, positive-strand RNA virus belonging to the genus *Alphacoronavirus* of the family *Coronaviridae* (2). The genome is approximately 28kb in length and encodes 4 structural (spike, envelope, membrane, and nucleocapsid) and 16 non-structural proteins (Nsp1-16), as well as an auxiliary protein (ORF3) used for virus replication (2, 3). Among these viral proteins, the spike (S) protein on the virion surface plays a crucial role in the entry of the virus into host cells and the induction of antibody responses (4, 5). The S protein undergoes post-translational modifications through the addition of a variable number of N-glycans (6). Glycosylation is essential for the proper folding, expression, and function of the coronavirus S protein (7–9).

Glycosylation is one of the most important post-translational modifications of proteins, with N-glycosylation being the predominant type. N-glycosylation occurs when N-acetylglucosamine (GlcNAc) in an oligosaccharide covalently binds to the polypeptide chain via an N-glycoside linkage to the amide nitrogen of an asparagine residue within the consensus NXT/S motif (10). The oligosaccharyltransferase (OST) complex is responsible for the formation of these glycosidic linkages in the lumen of the endoplasmic reticulum (ER) (11, 12). Mammalian cells express two OST complexes, each featuring different catalytic subunits (STT3A or STT3B) assembled with a shared set of non-catalytic subunits (ribophorin I, ribophorin II, OST48, DAD1, and OST4), as well as complex-specific subunits associated with the STT3B complex (MAGT1 or TUSC3) (13–15). Additionally, the extension of glycans requires the coordinated action of another glycosidase, which catalyzes the hydrolysis of glycosidic linkages (16, 17). Previous studies have shown that viral infections can be reduced with inhibitors of ER α-glucosidase (18, 19), which are essential enzymes for the maturation of N-glycan structures.

Glycosylation of viral envelope proteins is the main determinant of viral infection of target cells. In the case of SARS-CoV-2, blocking N and O-glycan biosynthesis during the elaboration of the SARS-CoV-2 S protein reduces viral infectivity (20). Mutations in the N-glycosylation sites also decrease viral particle production (21–23). Additionally, glycosylation can facilitate the correct folding of proteins, which affects their transport and stability (24). During HIV-1 replication, a high mannose type gp160 is transported to the Golgi apparatus from the ER and cleaved by a furin-like protease in the late Golgi to its mature gp120 and gp41 proteins, which remain associated (25). It has been reported that the removal of Asn332 in gp41 can disrupt the proteolytic processing and the transportation of gpl60 (26). Additionally, the absence of N-glycosylation on the surface of PEDV significantly reduces its infectivity (27), yet the specific functional role of the host’s N-glycosylation pathways during PEDV infection remains unclear.

Given the pivotal roles of protein N-glycosylation in viral biology, we first confirmed that the host glycosylation process is essential for PEDV replication using various glycosylation inhibitors. Subsequently, by knocking down the expression of STT3A and STT3B, the catalytically active subunits of the OST complex, we established that the STT3B-OST complex is preferentially required for PEDV replication. Together, these findings suggest that the host STT3B protein enhances viral replication through the N-glycosylation modification of the PEDV S protein.

## RESULTS

### N-glycosylation inhibitors are effective against PEDV infection

**Fig 1:**
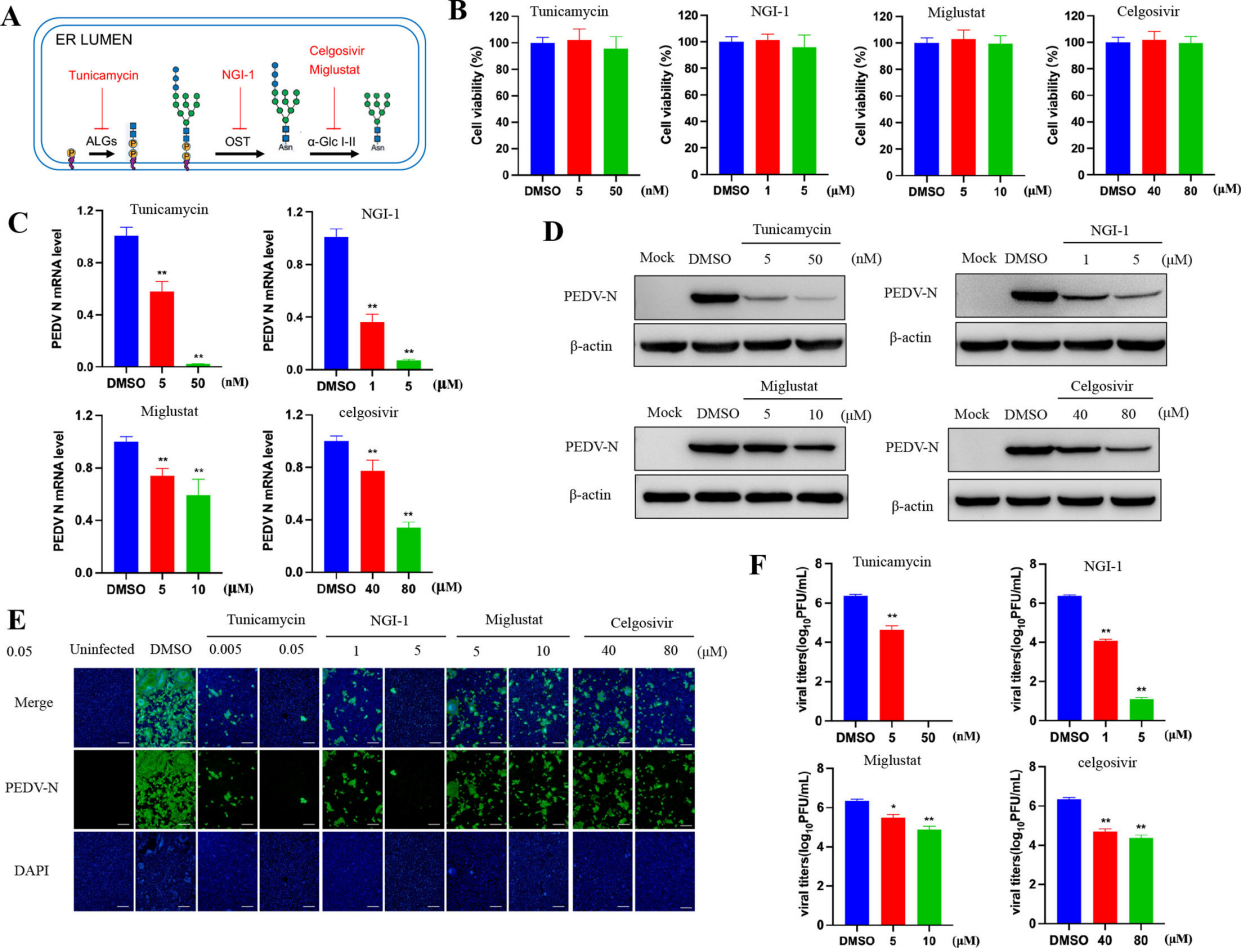
Glycosylation inhibitors reduce PEDV replication in Vero cells. (**A**) Schematic of the key steps in the N-glycosylation pathway targeted in this study. (**B**) Viability of Vero cells treated with the indicated concentrations of glycosylation inhibitors (tunicamycin at 5 and 50nM, NGI-1 at 1 and 5µM, miglustat at 5 and 10µM, and celgosivir at 40 and 80µM) for 24hours. (**C–F**) Vero cells pretreated for 1hour with inhibitors and infected or not (uninfected) with PEDV (MOI of 0.01, 20hours) in a fresh medium containing inhibitors. (**C**) Relative PEDV N mRNA levels determined by RT-qPCR and expressed relative to that in dimethyl sulfoxide (DMSO)-treated cells. GAPDH was used as the internal loading control. (**D**) Western blot of N-protein in cells infected with PEDV and treated with the indicated concentrations of glycosylation inhibitors or DMSO. (**E**) Immunoﬂuorescence assay (IFA) images of Vero cells infected with PEDV and treated with indicated inhibitors. Viral N-protein is shown in green, and the nuclei are blue. Scale bars, 500µm. (**F**) Culture supernatants were collected for viral titration. No infectious virus particles were detected in the supernatant of cells treated with tunicamycin at a concentration of 50nM. The data are presented as the means ± SDs. The asterisks indicate significant differences (*P < 0.05; **P < 0.01).

Given the potentially significant roles of protein N-glycans, we hypothesized that inhibiting the host N-glycosylation pathway would interfere with the development of PEDV infection, potentially affecting the normal production of viral proteins and virions and hampering their ability to infect additional host cells. Several compounds suppressing the functions of the essential N-glycosylation steps were used: (i) tunicamycin to block the formation of the N-glycan intermediate GlcNAc2-dolichol phosphate; (ii) NGI-1, which inhibits oligosaccharyltransferase catalytic subunit STT3 (isoforms A and B), to abolish transfer of N-glycan precursors to proteins in the ER; and (iii) the inhibitors of α-glucosidase I and II (miglustat and celgosivir) to prevent the deglycosylation of immature N-glycans attached to proteins (Fig. 1A).

The cytotoxicity of the inhibitors was initially assessed to rule out poor metabolism or toxicity as the primary cause of the decrease in infection; as shown in Fig. 1B, the cell viability of Vero cells remained above 95% at the selected concentrations. We then investigated whether these inhibitors provide protection against the virus. Vero cells were treated with the inhibitors for 1hour prior to PEDV infection, and the cells were collected at 20 hours post-infection (hpi) to assess PEDV production. The results demonstrated a dose-dependent antiviral activity of these inhibitors against PEDV reflected by RT-qPCR, Western blotting, IFA, and titration analysis (Fig. 1C through F). In particular, we found that the most effective strategies for reducing viral infection targeted the early steps of the N-glycosylation pathway; specifically, the inhibition of GlcNAc2-dolichol formation by tunicamycin and the direct inhibition of the OST complex using NGI-1 resulted in the greatest reductions in virus infection.

**Fig 2:**
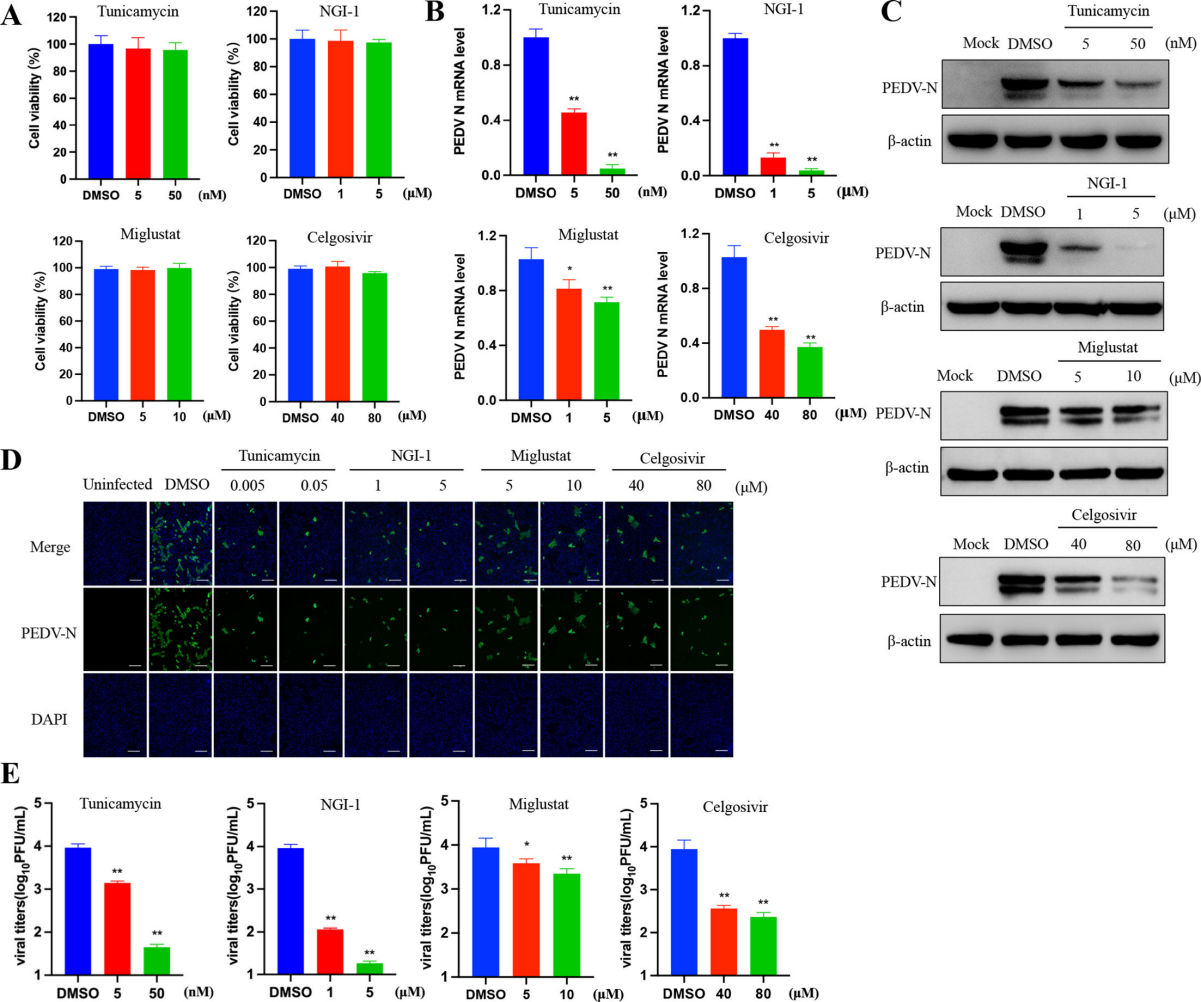
Glycosylation inhibitors reduce PEDV replication in LLC-PK1 cells. (**A**) Viability of LLC-PK1 cells pretreated with the indicated concentrations of glycosylation inhibitors and incubated for 20 hours in a medium containing inhibitors. (**B–E**) LLC-PK1 cells were pretreated with inhibitors for 1 hour and infected with PEDV for 1 hour at 37°C. After incubation for a total of 20 hours in a fresh medium containing inhibitors, relative PEDV N mRNA levels were determined by RT-qPCR (**B**). (**C**) Western blot of the PEDV N protein infected with PEDV and treated with inhibitors at 20 hpi. (**D**) IFA images of LLC-PK1 cells (PEDV-infected and inhibitors-treated) at 20 hpi. The PEDV N protein is green, and the nuclei are blue. Scale bars, 500 µm. (**E**) The culture supernatants were collected for viral PFU assay. The data are presented as the means  ±  SDs. The asterisks indicate significant differences (*P < 0.05; **P < 0.01).

Furthermore, the anti-PEDV activity of these four glycosylation inhibitors was explored in porcine renal epithelial cells (LLC-PK1). Cell viability analysis showed that these inhibitors exhibited little cytotoxicity on LLC-PK1 cells at various concentrations (Fig. 2A). The results revealed that these four inhibitors significantly decreased the PEDV N mRNA level, N protein expression, and viral titer in a concentration-dependent manner by using RT-qPCR, Western blot, IFA, and titration analysis (Fig. 2B through E). These results demonstrated that inhibition of the glycosylation process is effective in suppressing PEDV infection *in vitro*.

### STT3B contributes to PEDV replication

**Fig 3:**
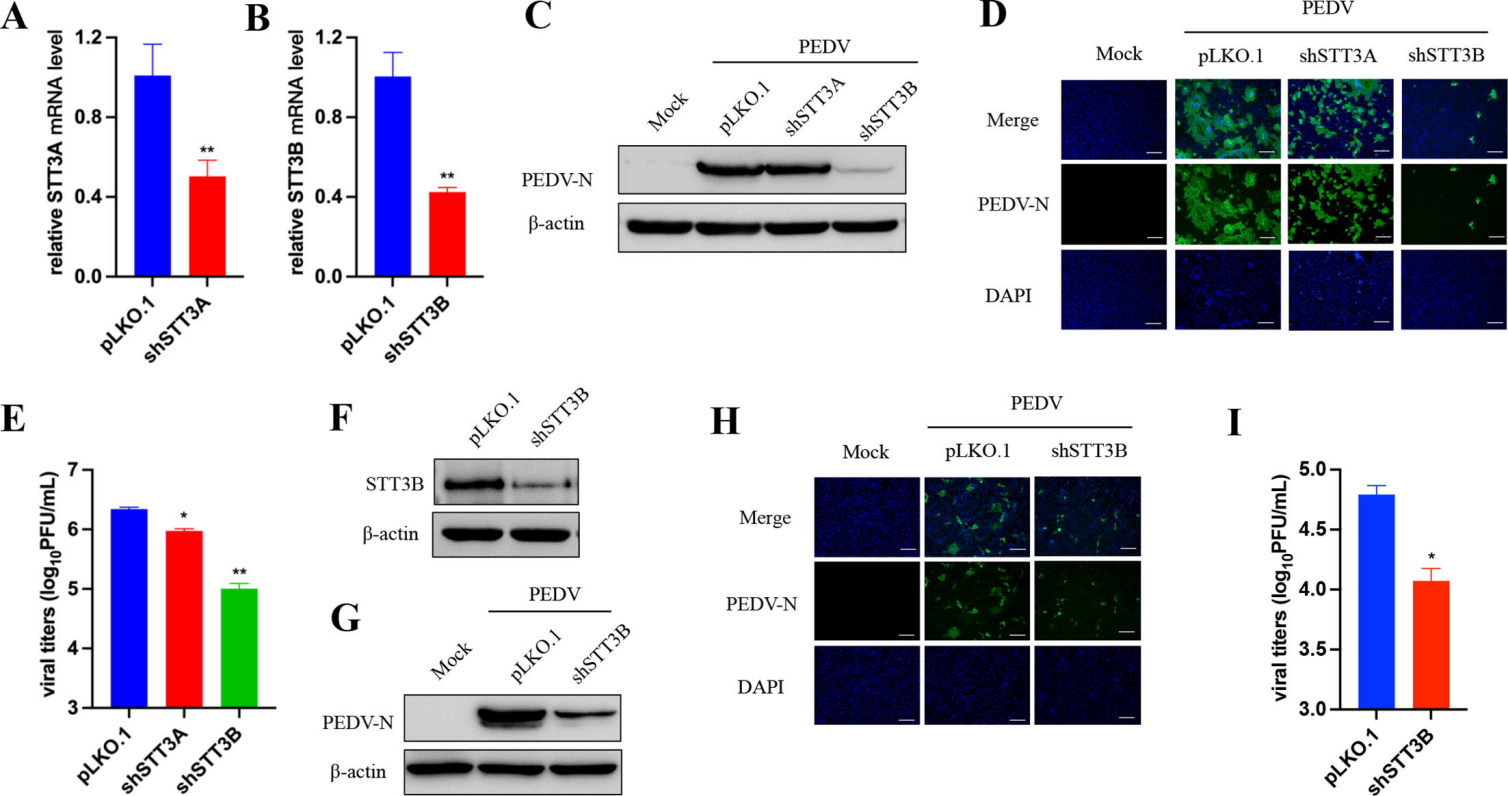
STT3B contributes to PEDV replication. (**A and B**) RT-qPCR analysis of Vero cells infected with STT3A, STT3B, or pLKO.1 control shRNA lentivirus. Data are normalized with pLKO.1 control shRNA. Western blot (**C**) and IFA (**D**) of N expression at 20 hours post-PEDV infection at an MOI of 0.01. (**E**) Supernatants were collected, and PFU assay was measured at 20 hpi. (**F**) Western blot analysis of lysates from LLC-PK1 cells infected with STT3B or pLKO.1 control shRNA lentivirus. β-actin was used as the loading control. Western blot (**G**), IFA (**H**), and virus titration (**I**) analysis of the effect of STT3B knockdown on PEDV replication in LLC-PK1 cells. Scale bars, 200 µm. The data are presented as the means ± SDs. *P < 0.05; **P < 0.01. All data are representative of three independent experiments.

Next, we investigated whether the genetic ablation of key glycosylation-modifying enzymes could reduce PEDV replication. STT3A and STT3B, two isoforms of the OST complex, exhibit distinct enzymatic properties, and their differential utilization may result in different glycosylation patterns (14). Thus, we assessed PEDV replication after knocking down STT3A or STT3B isoforms in Vero cells. Using the shRNA system, we generated STT3A and STT3B knockdown cells (Fig. 3A and B) and explored their roles in PEDV propagation. As shown in Fig. 3C and D, the PEDV N protein level was significantly downregulated in STT3B knockdown cells, as detected by Western blot and IFA. Additionally, progeny virus production was markedly reduced (Fig. 3E). In contrast, the knockdown of STT3A in Vero cells had a minimal effect on PEDV replication. Furthermore, the role of STT3B in viral replication was confirmed in LLC-PK1 cells, where similar results were observed through Western blot, immunostaining analysis, and titration assays after 20hours of infection (Fig. 3F through I). Together, these findings demonstrate that STT3B promotes PEDV replication.

### STT3B affects S protein glycosylation formation and subcellular localization

**Fig 4:**
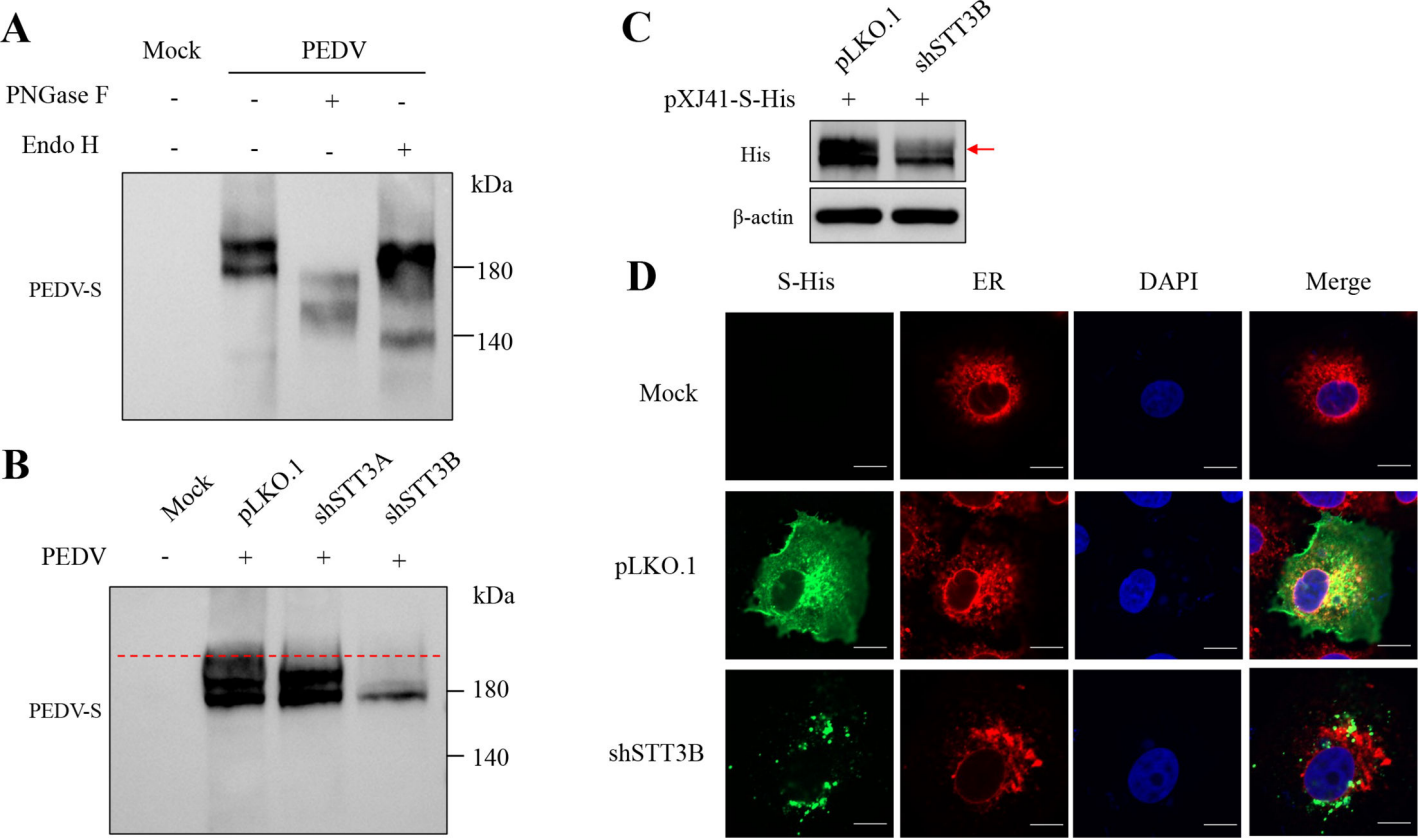
STT3B affects S protein N-glycosylation and subcellular localization. (**A**) Virions were untreated or treated with PNGase F or Endo H. The blot was incubated with mouse monoclonal antibody anti-S protein. (**B**) Western blot of S protein in Vero cells after knockdown of STT3A or STT3B. (**C**) Western blot analysis of exogenously expressed S protein in Vero cells with STT3B knockdown. (**D**) Intracellular localization of the S protein in WT and shSTT3B Vero cells. The S protein was stained with anti-His-tag primary antibodies and AlexaFluor488-conjugated secondary antibodies. The ER was visualized using anti-calreticulin antibodies and AlexaFluor594-conjugated secondary antibodies. Scale bars, 10 µm.

Given the importance of coronavirus S protein glycosylation for viral replication, we examined the effect of STT3B on S protein N glycosylation. N-glycans can be classified into three types: high mannose, complex, and hybrid, based on their degree of processing. Endoglycosidase H (Endo H) cannot cleave complex N-glycans, while N-glycosidase F (PNGase F) can (28). To investigate the glycosylation and maturation of the S protein, the virions were treated with PNGase F and Endo H (Fig. 4A). After treatment with PNGase F, the full-length S bands shifted, indicating the expected N-linked glycosylation of the protein. To assess the maturation status of the glycosylated proteins, we treated the purified virions with Endo H. The S protein’s resistance to this treatment indicated the presence of complex N-glycosylation.

Next, to determine whether STT3 isoforms play a role in glycosylation, we used Vero cells with knockdowns of STT3A or STT3B isoforms and assessed S protein expression in the context of viral infection. In shSTT3A cells, the viral S protein exhibited a glycosylation pattern similar to that of control cells, potentially reflecting the compensatory function of STT3B in these cells. However, knocking down STT3B dramatically reduced S protein glycosylation (Fig. 4B). We then transfected an S protein overexpression plasmid into STT3B-knockdown cell lines and observed that STT3B knockdown significantly reduced the glycosylation level of the S protein (Fig. 4C). Confocal experiments observed that the knockdown of STT3B altered the subcellular localization of the S protein compared to control cells (Fig. 4D). This finding supported the idea that STT3B-mediated N-glycosylation of the S protein enhances PEDV infection.

### STT3B interacts with PEDV S protein

**Fig 5:**
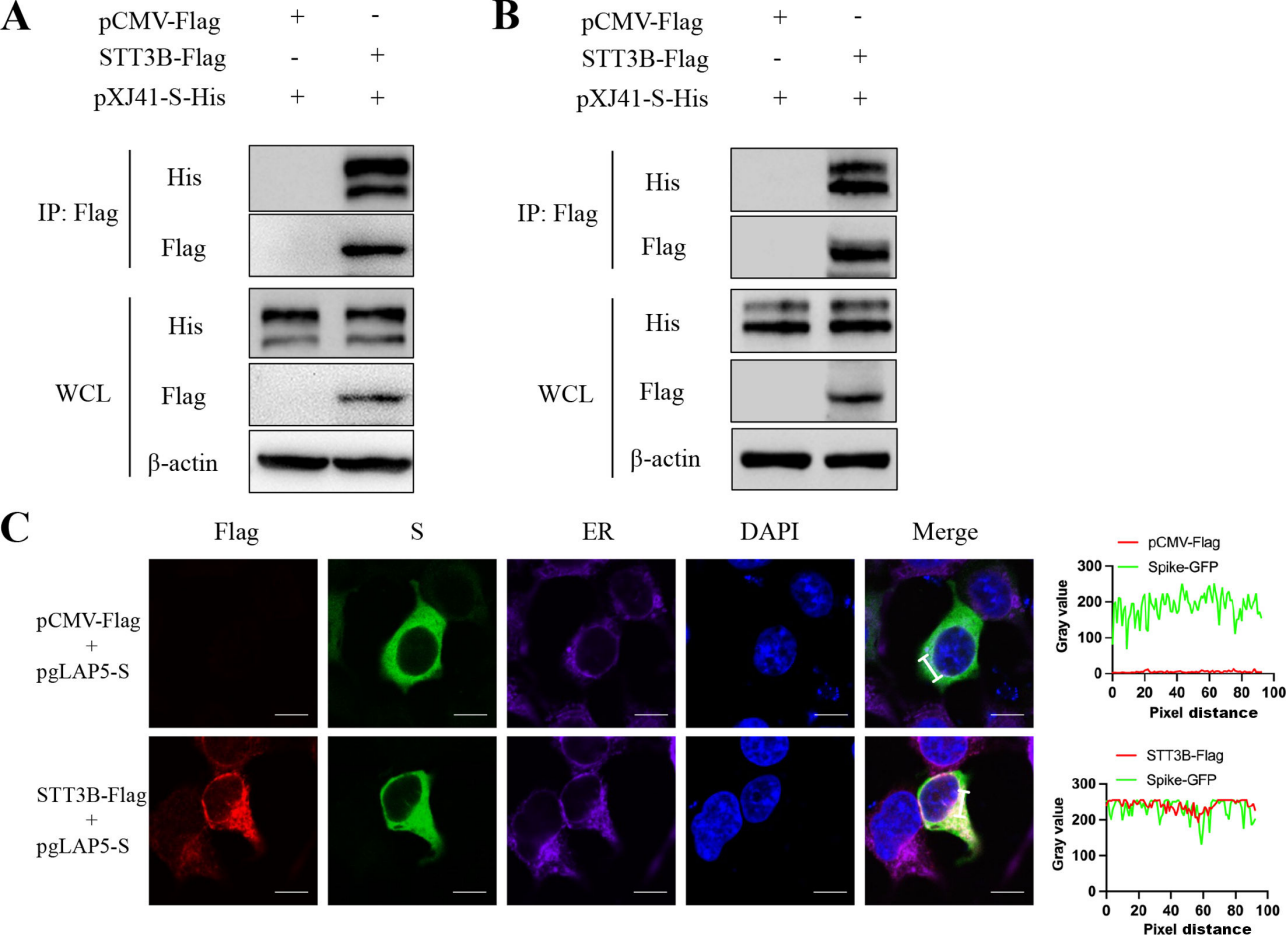
STT3B interacts with the PEDV S protein. (**A and B**) Co-IP assay demonstrates the binding of STT3B-Flag with the PEDV S protein. HEK293T (**A**) and LLC-PK1 (**B**) cells were co-transfected with STT3B-Flag or pCMV-Flag and PEDV S-His constructs. Cell lysates were incubated with anti-Flag magnetic beads. Immunoprecipitated samples were blotted with Flag and His antibody. (**C**) Immunofluorescence of STT3B and PEDV S in HEK293T cells. Cells were stained with Flag (red) and ER (purple) antibodies prior to analysis by confocal microscopy. The cell nuclei were stained with DAPI (blue). Line profiles corresponding to the white lines show colocalization. Scale bars, 10 µm.

To elucidate the mechanisms by which STT3B modifies S protein N-glycosylation, we investigated the interaction between STT3B and the S protein. The plasmid encoding Flag-tagged STT3B and His-tagged S protein was co-transfected into HEK-293T and LLC-PK1 cells. Co-immunoprecipitation (Co-IP) assays demonstrated that STT3B coprecipitated with the S protein (Fig. 5A and B). We then monitored the colocalization of STT3B and the S protein using confocal microscopy. Since it has been reported that STT3B is localized in the ER membrane (10), we simultaneously stained for the ER marker calreticulin. As indicated by fluorescence intensity, STT3B was highly colocalized with the S protein (Fig. 5C). The results suggest that STT3B may modify S protein N-glycosylation by interacting with the S protein and influencing its localization in the ER.

### The catalytic oligosaccharyltransferase activity of STT3B is required for S-protein glycosylation

**Fig 6:**
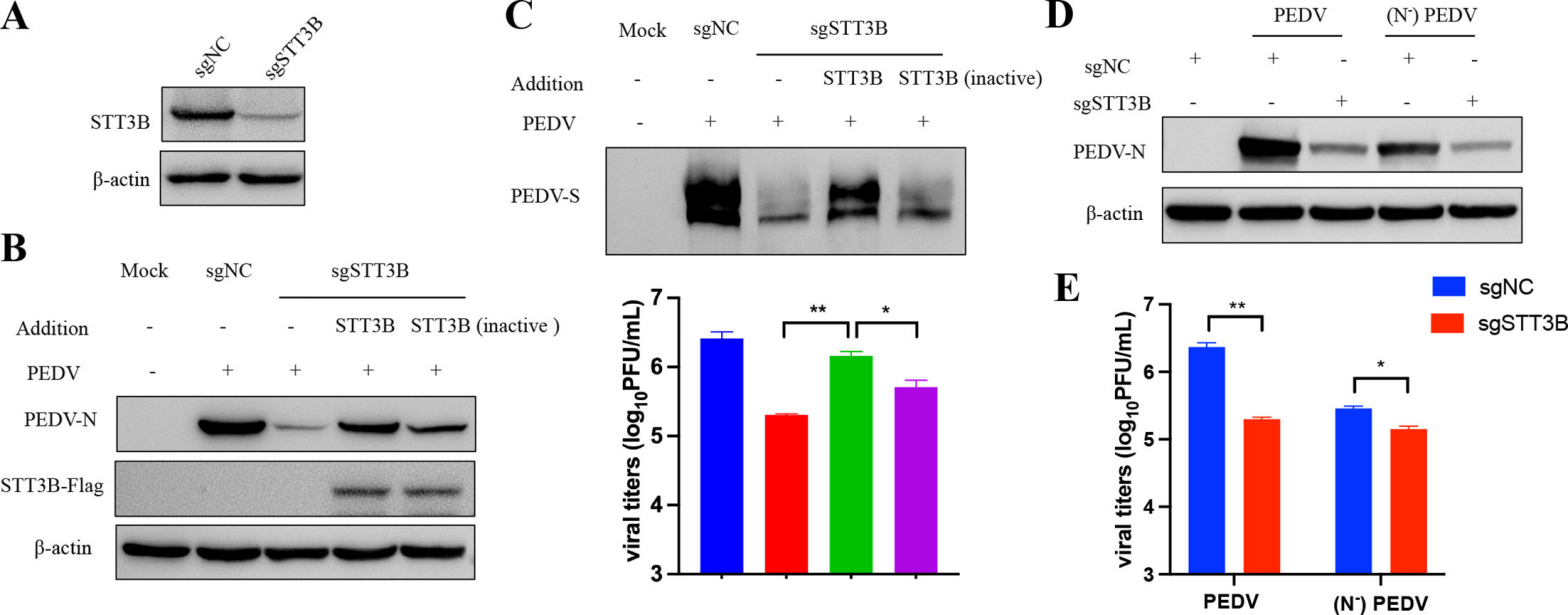
The catalytic activity of STT3B is essential for PEDV replication. (**A**) Western blot analysis of lysates from Vero cells transduced with STT3B or non-targeting control pLentiCRISPRv2 sgRNA lentivirus. β-actin served as the loading control. (**B**) STT3B KO cells transfected with STT3B WT or catalytically dead mutant (STT3B inactive) plasmid were infected with PEDV (0.01 MOI) at 24hours post-transfection. PEDV N protein in cells was analyzed by immunoblotting assay. (**C**) Cells were harvested from Fig. 5B and subjected to Western blot analysis with antibodies specific to the S protein (upper). Virus titers in supernatants were quantified by plaque assay (lower). (**D and E**) STT3B contributes to viral replication by influencing the formation of glycosylation of S protein. (**D**) STT3B KO cells and control cells were infected with wild-type or N-glycan-deficient (N^−^PEDV) PEDV for 20 hours. Detecting the load of PEDV N protein using Western blot. (**E**) Virus titers in supernatants were quantified by plaque assay. Bars indicate mean values, error bars represent ±standard deviations (SD), and the asterisks indicate significance (*P  <  0.05; **P  <  0.01).

Previously reported STT3B-OST has the capacity to mediate posttranslational modification of skipped glycosylation sites in unfolded proteins, and STT3B in oligosaccharyltransferases is necessary for the transfer of glycans to asparagines on target substrates (12). The conserved WWD motif of STT3B has been demonstrated to be essential for sequence recognition; mutation of WWDYG motif to WAAYG inactivates oligosaccharyltransferase activity (29, 30). To investigate the role of enzymatic activity of STT3B in PEDV replication, we utilized the CRISPR/Cas9 system (31) to knockout STT3B in Vero cells (Fig. 6A). Next, we overexpressed Flag-tagged wild-type (WT) or catalytically inactive STT3B in STT3B knockout Vero cells and infected them with PEDV after 20hours. The virus infection was assessed by Western blot and titration assays. As expected, PEDV replication decreased with the depletion of endogenous STT3B. However, overexpression of WT STT3B in the knockout cells restored PEDV replication nearly to WT levels, while the catalytically inactive STT3B only partially restored replication (Fig. 6B and C). We also noted that S protein glycosylation was affected by the loss of STT3B enzyme activity (Fig. 6C). These results indicate that the oligosaccharyltransferase activity of STT3B is essential for facilitating PEDV replication.

To investigate whether the STT3B protein regulates virus replication through glycosylation of the S protein, we utilized N-glycan-deficient viruses for validation. Initially, Vero cells were treated with the glycosylation inhibitor NGI-1 and subsequently infected with PEDV to generate N-glycan-deficient progeny viruses. We then infected both wild-type and STT3B-deficient Vero cells with either wild-type or N-glycan-deficient viruses at an MOI of 0.01. Western blot and titer assays demonstrated that, in comparison to wild-type PEDV, the absence of STT3B had a minimal effect on the replication of N-glycan-deficient viruses (Fig. 6D and E). The results indicate that STT3B promotes viral replication by modulating the glycosylation of the S protein.

## DISCUSSION

PEDV is one of the major causes of diarrhea in piglets, which is associated with a high mortality rate (32). Studies to understand its pathogenesis and replication cycles are urgently needed. It has been reported that cells with altered N-glycosylation release fewer virions, and the absence or abnormality of protein N-glycosylation in these viruses further affects their invasion, rendering a proportion of virions noninfectious to subsequent cells (33–35). Although defects in N-glycosylation have been shown to affect the infectivity of progeny PEDV, previous studies predominantly used kifunensine, an inhibitor of α-mannosidases in the Golgi apparatus, which specifically disrupts the later stages of the glycosylation process (27). To gain a deeper understanding of how host glycosylation pathways influence viral replication, we investigated the effects of glycosylation alterations within the ER on PEDV infection. In this work, we found that tunicamycin and NGI-1, targeting early steps of the N-glycosylation pathway, led to the strongest reductions in the production of infectious particles. Both the ER α-glucosidase I/II inhibitors miglustat and celgosivir were also successful in reducing PEDV infections, although not as effective as the earlier pathway inhibitors. Moreover, we identified that knockdown of STT3B, but not STT3A, specifically affects PEDV infection in Vero cells, as previously reported in SARS-CoV-2 (36). Their different substrate specificities may explain this difference between subtypes (37). Inhibition of N-glycosylation was tested here to be protective against PEDV infection, but further experiments are needed to explore which stage of PEDV replication is specifically affected by the inhibition of host N-glycosylation.

Coronavirus S protein plays a critical role in the viral life cycles. It facilitates viral entry via binding to the virus receptor at the extracellular membrane or promoting membrane fusion. Coronavirus S protein is first translated into the ER lumen and then undergoes a series of folding and modification processes along the secretory pathway to achieve a mature and functional conformation. Among these processes, N-glycosylation in the ER lumen is the most dominant modification of PEDV S protein. Blocking N-linked glycosylation of S protein by means of glycosylation inhibitors impedes viral entry (20). STT3A and STT3B are responsible for this modification, where they catalytically transfer a preassembled oligosaccharide to the asparagine residue of a nascent polypeptide (38). The VSV-G, influenza HA, and HIV-1 gp160 proteins have been reported to have different dependencies of STT3A and STT3B on N-glycosylation, suggesting that different viruses utilize different modification mechanisms (39), although the full glycosylation of polypeptides involves the cooperation of the two isoforms (11). Our study showed that STT3B knockdown caused hypo-glycosylation of S protein in the situation of viral infection, while STT3A knockdown did not have such an effect. This phenotype indicated that the PEDV S protein is preferentially modified by STT3B-OST isoforms. Further studies are needed to deeply clarify the physiological significance of this host glycosylation strategy.

It is important to point out that our study has limitations. STT3B knockdown cells expressing a catalytically inactive STT3B mutant were still able to support a moderate level of PEDV replication compared to knockdown cells, although at lower levels than those rescued with WT STT3B rescue. This suggests that the STT3B-OST complex, which lacks oligosaccharyltransferase activity, also retains other functions to support PEDV infection. This phenomenon may be due to the impact of STT3B deficiency affecting the expression of MAGT1 and its paralog TUSC3 in the STT3B complex (13). Oxidoreductase activity of MAGT1 and TUSC3 is required for N-glycosylation of cysteine proximal receptor sites in glycoproteins (40, 41). However, further experiments are necessary to demonstrate the effect of this hypothesis on PEDV replication.

In summary, we have identified that the host glycosylation pathway could be an effective target for blocking PEDV infection by using four inhibitors of glycosylation enzymes in the ER. PEDV utilizes host glycosylation-modifying enzymes to accomplish post-translational modification of its S protein, in which the STT3B-OST complex plays an important role. Our study promotes a more comprehensive understanding of PEDV infections and provides new evidence for the development of future antiviral drugs.

## MATERIALS AND METHODS

### Cell lines and viruses

Vero cells (ATCC CRL-1586) and HEK293T cells (ATCC CRL-3216) were maintained in Dulbecco’s modified Eagle’s medium (DMEM) (Gibco, USA) supplemented with 10% fetal calf serum (Gibco, USA) and 50 IU/mL penicillin/streptomycin (Corning, USA). LLC-PK1 cells (ATCC CL-101) were cultured in MEM (Gibco, USA). All the cells were incubated at 37°C in a humidified incubator with 5% CO_2_. The PEDV variant strain MSCH (GenBank accession no. MT683617, G2b subtype) was separated and maintained in our laboratory and passaged in Vero cells with 6 µg/mL trypsin (Sigma, USA) (42).

### Antibodies and N-glycosylation inhibitors

Rabbit anti-STT3B antibody, rabbit anti-Calnexin antibody, mouse anti-His antibody, and mouse anti-β-actin antibody were purchased from Proteintech (USA). Mouse anti-anti-Flag antibody was purchased from Abmart (China). Mouse anti-PEDV N protein and S protein monoclonal antibodies were produced in our laboratory. Horseradish peroxidase-labeled rabbit or mouse secondary antibodies were purchased from Jackson (USA). Cells were treated with either dimethyl sulfoxide (DMSO) or several N-glycosylation inhibitors (DMSO final concentration never exceeding 2%). Tunicamycin, NGI-1, miglustat, and celgosivir were purchased from MCE (USA).

### Plasmids and molecular cloning

The STT3B-3xFlag expression vector was purchased from Solarbio (China). Oligosaccharyltransferase activity mutation plasmid of STT3B was generated by PCR-directed site mutagenesis. This cDNA sequence of the S protein of PEDV was cloned into the pXJ41 vector fused with a C-terminal His-tag or cloned into pgLAP5 plasmid to achieve fusion with an enhanced green fluorescent protein sequence. pLKO.1-TRC vector (Addgene) was digested by *EcoRI* and *AgeI* restriction enzyme (Thermo Scientific) to prepare backbone and then annealed shRNA oligo was ligated with backbone by T4 DNA ligase (Thermo Scientific) to construct knockdown shRNA. plentiCRISPRv2 vector (Addgene) was digested by *BsmBI* restriction enzyme (NEB) and ligated with sgRNA. The primers used for molecular cloning are listed in Table 1.

**TABLE 1:** Primers used in the study

Primer name	Sequence (5′ → 3′)	Usage
STT3B-mutant-F	GCGATCTGATAGCCATATGCTGCCCAGGACATGACCCGGGCAT	Construction of plasmid pCMV-STT3B inactive -3xFlag
STT3B-mutant-R	GCAGCATATGGCTATCAGATCGCTGGG	
S-His-F	ATAGAAGATTCTAGAGCTAGCATGCACCACCACCACCACCATC	Construction of plasmid pXJ41-S-His
S-His-R	GGTATCGATAAGCTTGATATCTCAATGGTGATGGTGATGGTGGTAAGGTTGAAGTCT	
Monkey shSTT3A-F	CCGGGGACGAATCATTGGAGGAACACTCGAGTGTTCCTCCAATGATTCGTCCTTTTTG	Construction of plasmid pLKO.1-shSTT3A (monkey)
Monkey shSTT3A-R	AATTCAAAAAGGACGAATCATTGGAGGAACACTCGAGTGTTCCTCCAATGATTCGTCC	
Monkey shSTT3B-F	CCGGGCAGTATCTGAGAGACCGATTCTCGAGAATCGGTCTCTCAGATACTGCTTTTTG	Construction of plasmid pLKO.1-shSTT3B (monkey)
Monkey shSTT3B-R	AATTCAAAAAGCAGTATCTGAGAGACCGATTCTCGAGAATCGGTCTCTCAGATACTGC	
Porcine shSTT3B-F	CCGGGACGCGTACCTGCTGTATATACTCGAGTATATACAGCAGGTACGCGTCTTTTTG	Construction of plasmid pLKO.1-shSTT3B (porcine)
Porcine shSTT3B-R	AATTCAAAAAGACGCGTACCTGCTGTATATACTCGAGTATATACAGCAGGTACGCGTC	
Monkey sgSTT3B-F	CACCGAATGGCTAATAGAACTACGT	Construction of plasmid pLentiCRISPRv2-sgSTT3B (monkey)
Monkey sgSTT3B-R	AAACACGTAGTTCTATTAGCCATTC	
PEDV N-F	TTCTTGTTTCACAGGTGGATG	RT-PCR detection for PEDV N
PEDV N-R	GCTGCTGCGTGGTTTCA	
Monkey GAPDH-F	CCTTCCGTGTCCCTACTGCCAAC	RT-PCR detection for GAPDH (monkey)
Monkey GAPDH-R	GACGCCTGCTTCACCACCTTCT	
Porcine GAPDH-F	TGGTGAAGGTCGGAGTGAAC	RT-PCR detection for GAPDH (porcine)
Porcine GAPDH-R	AGTGGAGGTCAATGAAGGGG	
Monkey STT3A-F	AATACTCCAGAGGATGCGAAGG	RT-PCR detection for STT3A (monkey)
Monkey STT3A-R	ATGGTTCGGTTTGCCATAGC	
Monkey STT3B-F	AAGTGAACACATGGCAGCTG	RT-PCR detection for STT3B (monkey)
Monkey STT3B-R	TCGGTCTCTCAGATACTGCAA	

### Cytotoxicity assay

The test compounds were added to the corresponding cells and incubated for 24–48 hours at 37°C. The assessment of cellular viability was conducted utilizing an enhanced Cell Counting Kit-8 (CCK-8; Beyotime, China) following the manufacturer’s instructions. An equal volume of DMSO was used as the control.

### Western blot analysis

Cells were lysed with radioimmunoprecipitation assay (RIPA) lysis buffer (Beyotime, China) on ice for 30 min, separated by SDS–PAGE, and transferred to a nitrocellulose membrane. The membrane was then incubated in blocking buffer (10% nonfat milk in PBST) for 2 hours at room temperature, washed with PBST, and then incubated with the corresponding primary antibodies overnight at 4°C. After incubation with the corresponding secondary antibodies for 1 hour at RT and treatment with an enhanced chemiluminescence kit (Tanon, China), the specific bands were analyzed using a Tanon 5200 chemiluminescence imaging system (Tanon, China).

### RT-qPCR

Cells were washed with PBS once, and RNA was extracted from the cells using a Total RNA Kit I (Omega Bio-Tek, USA) following the manufacturer’s instructions. For reverse transcription, 1 µg total RNA was reverse transcribed using HiScript qRT SuperMix (Vazyme, China). Reactions were performed by an ABI QuantStudio 6 System (Applied Biosystems, USA) using AceQ qPCR SYBR Green Master Mix (Vazyme, China) according to the instructions. Relative expression levels of the target genes were calculated using the comparative cycle threshold method. Host and PEDV were normalized relative to the housekeeping gene. The primers used for RT-qPCR are listed in Table 1.

### Lentivirus production and transduction

Lentiviruses were produced by transient transfection of the packaging plasmids pMD2.G (12259, Addgene), psPAX2 (12260, Addgene), and pLKO.1-shRNA or pLentiCRISPRv2-sgRNA with polyethylenimine linear transfection reagent (Yeasen, China) into HEK293T cells. The supernatant was changed 12hours post-transfection. Supernatants were collected at 48 and 60hours after transfection and frozen at −80°C. Cells were transduced with lentivirus in the presence of 10µg/mL polybrene.

### Co-immunoprecipitation assay

Cells were transfected with plasmids for 24 hours and lysed with RIPA cell lysis buffer containing phenylmethanesulfonyl fluoride (PMSF) (protease inhibitor, Beyotime). Collected lysates were centrifuged and incubated on anti-Flag magnetic beads (Sigma, USA). This mix was washed with PBST resuspension in 50 mM glycine elution buffer (pH 2.8). Using specific antibodies, immunoblotting was done to examine the proteins.

### Immunoﬂuorescence assay and confocal microscopy

Immunostaining was performed on cells grown on 48-well plates or a 15 mm cell culture dish (Nest Biotechnology, China), after fixation with 4% paraformaldehyde for 10 min at room temperature and permeabilization in 0.1% Triton X-100 for 10 min, and blocked with 2% bovine serum albumin in PBS for 1 hour. Following incubation with the primary antibody at 4°C overnight, the cells were washed and incubated with the Alexa Fluor 488-conjugated secondary antibody (Proteintech, USA) for 1 hour. Finally, cells were stained with DAPI (Biosharp, China) to show the nucleus in blue for 10 min. After washing with PBST, cells were visualized with confocal microscopy (Nikon A1, Japan). Two or three channels were recorded sequentially or simultaneously while watching for potential overlaps among the different color signals.

### Plaque assays

Vero cells were cultured in 6-well plates and allowed to reach confluence overnight. Cells were incubated with 1 mL serially diluted supernatant containing PEDV for 2 hours at 37°C and 5% CO_2_ before being left in 2 mL overlay (DMEM, 1% low-melting agar, and 8 µg/mL trypsin) at 37°C and 5% CO_2_ for 72 hours. Cells were stained with crystal violet staining solution (20% ethanol solution, 2% formaldehyde, and 1% crystal violet) for manual plaque counting.

### Deglycosylation assay

Endo H (NEB, USA) and PNGase F (NEB, USA) treatment was performed according to the manufacturer’s recommendations. Briefly, lysates of transfected cells were mixed with denaturing glycoprotein buffer and heated at 100°C for 5 min. Subsequently, 20 units of PNGase F or Endo H were added to samples in a final volume of 20 µL. The reaction mixtures were incubated for 1 hour at 37°C before samples were used for Western blot analysis.

### Generation of N-glycan-deficient viruses

Vero cells were cultured in 6-well plates, with one set treated with the N-glycosylation inhibitor NGI-1 at a final concentration of 1 µM, and another set left untreated as a control. After a 4-hour incubation, the cells were infected with PEDV at an MOI of 0.01. One hour post-infection, the viral inoculum was removed, and the cells were replenished with a fresh culture medium containing 1 µM NGI-1. The viral supernatant was collected at 48 hours post-infection, subjected to three freeze-thaw cycles, and then analyzed for viral titer determination.

### Statistical analysis

The data from three individual experiments were assessed by one-way or two-way ANOVA with Tukey’s *post hoc* test for multiple comparisons (GraphPad Prism Software Inc, San Diego, CA, USA) and presented as mean ± SD (standard deviation). A *P*-value < 0.05 was considered statistically significant.

## Data Availability

The authors confirm that the data supporting the findings of this study are available within the article.
